# Nomogram model for the risk of insulin resistance in obese children and adolescents based on anthropomorphology and lipid derived indicators

**DOI:** 10.1186/s12889-023-15181-1

**Published:** 2023-02-07

**Authors:** Cao You-xiang, Zhu Lin

**Affiliations:** 1 Graduate Department, Guangzhou Sport University, Guangzhou, Guangdong Province China; 2 School of Sport & Health, Guangzhou Sport University, No. 1268, Guangzhou Avenue Middle, Tianhe District, Guangzhou City, Guangdong Province China

**Keywords:** Nomogram, Insulin resistance, Obese adolescents, Predictive model, Derived indicators

## Abstract

**Objective:**

This study aims to screen for measures and lipid-derived indicators associated with insulin resistance (IR) in obese children and adolescents and develop a nomogram model for predicting the risk of insulin resistance.

**Methods:**

A total of 404 eligible obese children and adolescents aged 10–17 years were recruited for this study from a summer camp between 2019 and 2021. The risk factors were screened using the least absolute shrinkage and selection operator (LASSO)-logistic regression model, and a nomogram model was developed. The diagnostic value of the model was evaluated by plotting the receiver operator characteristic curve and calculating the area under the curve. Internal validation was performed using the Bootstrap method, with 1000 self-samples to evaluate the model stability. The clinical applicability of the model was assessed by plotting the clinical decision curve.

**Results:**

On the basis of the LASSO regression analysis results, three lipid-related derivatives, TG/HDL-c, TC/HDL-c, and LDL-c/HDL-c, were finally included in the IR risk prediction model. The nomogram model AUC was 0.804 (95% CI: 0.760 to 0.849). Internal validation results show a C-Index of 0.799, and the mean absolute error between the predicted and actual risks of IR was 0.015. The results of the Hosmer–Lemeshow goodness-of-fit test show a good model prediction (χ^2^ = 9.523, P = 0.300).

**Conclusion:**

Three early warning factors, TG/HDL-c, TC/HDL-c, and LDL-c/HDL-c, were screened, which can effectively predict the risk of developing IR in obese children and adolescents, and the nomogram model has an eligible diagnostic value.

**Supplementary Information:**

The online version contains supplementary material available at 10.1186/s12889-023-15181-1.

## Introduction

Insulin resistance (IR) was the abnormal state of decreased insulin sensitivity in body tissues, such as liver or muscle, and the pathophysiological basis of several metabolic diseases[1, 2]. Obesity was a direct cause of IR, and the prevalence of IR was also significantly higher in obese children and adolescents (higher than 30%) than normal weight children and adolescents[3]. When IR occur, it will increase the risk of cardiovascular disease through various mechanisms[4, 5], and some studies also found that IR has a significant positive correlation with cancer[6, 7]. IR not only poses a risk to children’s physical health but also increased the incidence of type 2 diabetes mellitus and cardiovascular disease in adulthood[8, 9]. With the development of insulin resistance always accompanied by the decline of islet β-cell function. Unlike adults, the decline of islet β-cell function was more rapid in children, and with a faster rate of associated complications[8]. The current IR diagnosis was the high insulin normoglycemic clamp technique[10] or the homeostasis model assessment index of IR (HOMA-IR)[11], but these methods are all post hoc diagnostics and do not provide effective early warning. This also indirectly led to the high prevalence of IR in obese children and adolescents not receiving the timely preventive intervention. Therefore, early warning and prevention of the onset of IR in obese children were necessary.

Recently, some novel anthropometric derived indices, such as conicity index (CI) and body roundness index (BRI), have been found superior to conventional morphological indices in the prediction of IR[12–14]. In addition to anthropometric derived indices, lipid-related derived indices, such as Triglycerides(TG)/high-density lipoprotein cholesterol(HDL-c) ratio (TG/HDL-c), total cholesterol(TC)/HDL-c ratio (TC/HDL-c), and low-density lipoprotein cholesterol(LDL-c)/HDL-c ratio (LDL-c/HDL-c), also found be the effective predictors of IR and have a higher predictive diagnostic value than conventional lipid indices alone[15–18]. However, whether anthropometric-derived index or lipid-derived index, a single index has lower diagnostic efficacy in IR predictive model. In contrast, studies found that combined multi-indicator diagnosis usually possesses higher diagnostic effects than single-indicator models[19]. Therefore, it remains to be further investigated whether the predictive efficacy of IR can be improved by co-modeling with lipid-derived indices by measurement to better predict the risk of IR development.

Therefore, we screened novel anthropometric-derived and lipid-related indicators to establish a predictive model with diagnostic value for the development of IR in obese children and adolescents and provided a clinical reference for the prevention of IR.

## Participants and method

### Participants

**Fig. 1:**
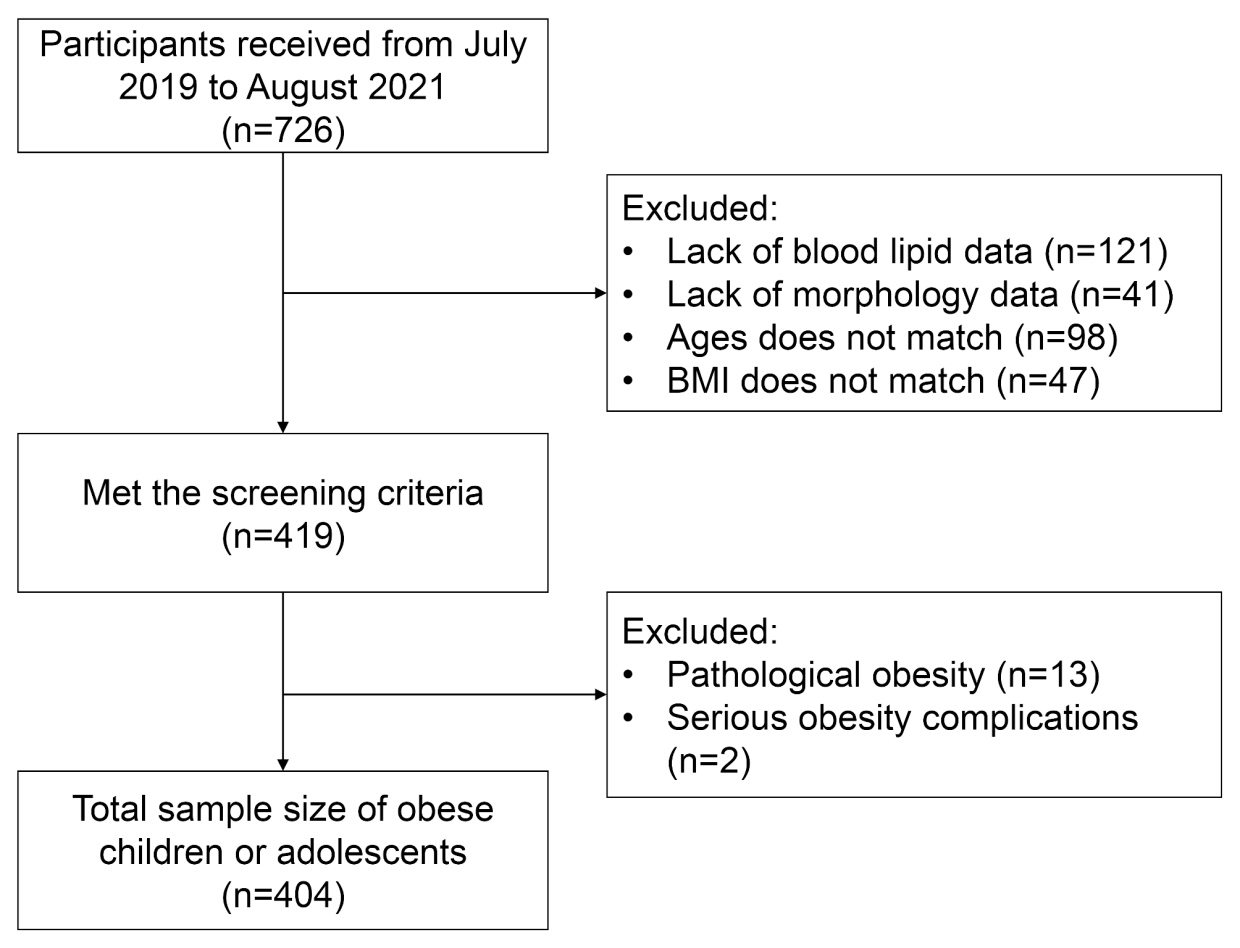
Participants Screened Flow

In this study, obese children and adolescents who participated in the Biggest Loser Training Camp between 2019 and 2021 were invited as the study population, and the screening process was shown in Fig. 1. The Biggest Loser Training Camp was a summer weight loss camp for obese children and adolescents. All the obese children and adolescents will participate in weight loss exercise training program and sleeping two to a dorm and eating together in one big dining hall. The screening criteria were as follows: (1) age between 12 and 17 years old, (2) normal intellectual development, (3) obesity identification criteria for Chinese children and adolescents[20], and (4) can cooperate with the researchers to complete relevant tests. The exclusion criteria were as follows: (1) pathological obesity [BMI ≥ 40 kg/m^2^[21]], (2) serious obesity complications [confirmed cardiovascular disease, obstructive sleep apnea, musculoskeletal problems, idiopathic intracranial hypertension[22, 23]], (3) being treated with medication for obesity or other any diseases (like hepatobiliary disease, chronic kidney disease), and (4) fasting plasma glucose (FPG) > 5.60 mmol/L. All the data included in this paper were collected before the weight loss program, and all participants and parents were informed of the possible risks associated with the experiment and signed an informed consent form before the experiment.

On the basis of Harrell’s guidelines[24], when the outcome is binary, the minimum value of the frequencies of the two response levels should be greater than 10 times the number of predictors. In our study, there were 10 predictors, thus the minimum sample size (IR group) is 100.

### Data measurement

The basic details of the subjects, such as their height, body weight (BW), age, gender, waist circumference (WC), and hip circumference (HC), were collected during fasting condition. The height was measured to the nearest 0.1 cm using a standard height meter (RuKe RK001, Zhejiang, China)[25], and the weight was measured to the nearest 0.1 kg on a digital scale (Inbody 370, Korea)[26]. The body mass index (BMI) was calculated by weight in kilograms divided by the square height in meters. Waist and hip circumferences were measured using an inelastic plastic fiber tape (RHOS, GC059, Guangdong, China) measure placed directly on the skin at the midpoint between the lower border of the rib cage and the iliac crest(for waist)[27] and at the maximum extension of the buttocks (for hip)[28]. To guarantee the quality control of the measurements, BW, height, HC and WC were measured twice by a single evaluator and average value was taken: The values of the technical error of measurement range from 1.0 to 2.0%. Before the study begins, a specialist in anthropometric measurements provided a theoretical and practical training to the evaluator, and dozens of children and adolescents were measured at the same time by the specialist and the evaluator to confirm that the measurements were accurate.

Fasting venous blood samples were collected in the early morning (the fasting time was more than 10 h). The supernatant was extracted after centrifugation at 4 °C (30 min at 1000 g) and stored at -80 ℃ for measured. The FPG was measured using the glucose oxidase method. The fasting insulin (FINs) was obtained using the electrochemiluminescence method, while the blood lipid (TC, TG, HDL-c, and LDL-c) was obtained using a fully automatic biochemical analyzer (Beckman, AU5800, Japan).

The IR diagnosis was based on the HOMA-IR level, and the criteria were as follows: HOMA-IR > 3 and HOMA-IR = FINs (µU/L)*FPG (mmol/L)/22.5[3].

The anthropomorphology derived indicators contained in this study were: body mass index(BMI), waist hip rate(WHR), waist height ratio(WHtR), CI[29], BRI[30], abdominal volume index(AVI)[31], body adiposity index(BAI)[32], tri-ponderal mass index(TMI)[33]. The calculation formula was shown in the supplementary file.

### Statistical analysis

The R (version 4.2.0, https://www.r-project.org/) and SPSS 25.0 (SPSS, Inc., Chicago, IL, United States) software were used for the statistical analysis of the data. The Kolmogorov–Smirnov method was used to test the normality of continuous variables. The data that follows normal distribution are expressed as mean ± standard deviation (mean ± SD). One-way analysis of variance (ANOVA) was used to analyze the differences between groups. The data that do not follow normal distribution are expressed as median (quartiles) [M (P25, P75)], and the Mann–Whitney U test was used to determine the differences between groups. Categorical variables are expressed as frequencies (percentages), and the chi-square test was used to compare the differences between groups.

Given the strong collinearity between the anthropometric and lipid-derived indicators in this study, the least absolute shrinkage and selection operator (LASSO) and multiple logistic regression were used to screen the LDL-c/HDL-c, TC/HDL-c, TG/HDL-c, and other body measurements and the lipid-related derivatives, and a nomogram model for the risk of IR was developed. The model discrimination was verified in terms of the receiver operating characteristic (ROC) and the area under the curve (AUC), Akaike information criterion (AIC), Bayesian information criterion (BIC). The calibration of the nomogram model was validated in terms of the calibration curve and the Hosmer–Lemeshow goodness-of-fit. Nomogram model validation was performed using the Bootstrap method with 1000 replicate samples for internal validation, and the consistency index (C-Index) was calculated using Harrell’s C-Index to verify the model prediction performance. An AUC or C-Index less than 0.65 indicates poor model discrimination, 0.65 to 0.75 indicates that the model has some discriminatory ability, and a value greater than 0.75 indicates that the model has a good discriminatory ability[34]. Finally, clinical decision curves were plotted to assess the clinical benefit of the nomogram[35].

## Results

### Participants characteristics

A total of 404 eligible obese children and adolescents (226 males) were included in this study, namely, 138 in the IR group and 256 in the no-IR group. No difference in age, height, and WHR was observed between the IR and no-IR groups, while the body weight, HOMA-IR, FPG, FINs, TC, TG, HDL-c, LDL-c, WHtR, and CI in the IR group were significantly different from those in the no-IR group (P < 0.05), as shown in Table 1.

**Table 1:** Baseline characteristics of the participants

	Total (n = 404)	IR Group (n = 138)	no-IRGroup (n = 266)	*P-value*
Male (%)	226(55.90%)	76(55.07%)	150(56.39%)	
Age (years)	14.00(12.00,15.00)	14.00(13.00,15.00)	14.00(12.00,15.00)	0.529
H(cm)	163.89 ± 9.22	164.76 ± 9.52	163.44 ± 9.04	0.175
BW(kg)	82.23 ± 16.34	84.77 ± 17.69	80.91 ± 15.47	0.024
BMI(kg/m^2^)	30.36 ± 3.87	30.92 ± 3.77	30.07 ± 3.90	0.037
WC(cm)	97.15 ± 10.40	98.74 ± 10.89	96.32 ± 10.07	0.026
HC(cm)	107.23 ± 8.91	108.64 ± 8.65	106.57 ± 8.97	0.027
WHR	0.91 ± 0.07	0.91 ± 0.07	0.90 ± 0.07	0.531
WHtR	0.59(0.55,0.63)	0.60(0.56,0.63)	0.58(0.55,0.62)	0.038
CI	0.61(0.55,0.69)	0.63(0.57,0.72)	0.61(0.55,0.68)	0.032
BRI	5.32 ± 1.23	5.46 ± 1.22	5.25 ± 1.23	0.096
AVI	21.73(19.56,24.40)	22.13(20.06,24.58)	21.47(19.35,24.13)	0.056
TMI	18.53 ± 2.19	18.76 ± 1.95	18.41 ± 2.29	0.126
BAI	28.36 ± 4.38	28.73 ± 4.24	28.17 ± 4.45	0.222
TC(mmol/L)	4.47 ± 1.09	4.98 ± 1.29	4.21 ± 0.87	<0.001
TG(mmol/L)	0.91(0.67,1.25)	1.23(0.88,1.80)	0.80(0.62,1.09)	<0.001
HDL-c(mmol/L)	1.26(1.08,1.47)	1.20(0.96,1.37)	1.30(1.12,1.50)	<0.001
LDL-c(mmol/L)	2.56 ± 0.71	2.89 ± 0.82	2.38 ± 0.58	<0.001
TG/HDL-c	0.72(0.49,1.09)	1.09(0.72,1.56)	0.60(0.44,0.88)	<0.001
TC/HDL-c	3.41(2.81,4.21)	4.12(3.35,5.09)	3.18(2.67,3.72)	<0.001
LDL-c/HDL-c	1.99(1.56,2.49)	2.40(1.89,3.08)	1.76(1.44,2.26)	<0.001
FPG(mmol/L)	4.62(4.07,5.11)	5.00(4.63,5.33)	4.35(3.97,4.88)	<0.001
FINs(µU/mL)	11.35(7.52,16.56)	20.60(16.19,26.31)	8.56(5.81,13.61)	<0.001
HOMA-IR	2.91 ± 2.42	5.25 ± 2.81	1.70 ± 0.73	<0.001

*AVI: Abdominal volume index; BAI: Body Adiposity Index; BMI: body mass index;BRI: Body Roundness Index; BW: Body Weight; CI: Conicity Index; FPG: Fasting Plasma Glucose; FINs: Fasting Insulin; HOMA-IR: Homeostasis Model Assessment index of Insulin Resistance; H: Height; HC: Hip Circumference; HDL-c: High-density Lipoprotein Cholesterol; LDL-c/HDL-c:The ratio of LDL-c and HDL-c; WC: Waist Circumference; WHR: Waist Hip Rate; WHtR: Waist Height Rate; TC: Total cholesterol; TMI: Tri-Ponderal Mass Index; TG: Triglycerides; TG/HDL-c: The ratio of TG and HDL-c; TC/HDL-c:The ratio of TC and HDL-c.*

### Screening of IR-related risk factors

**Fig. 2:**
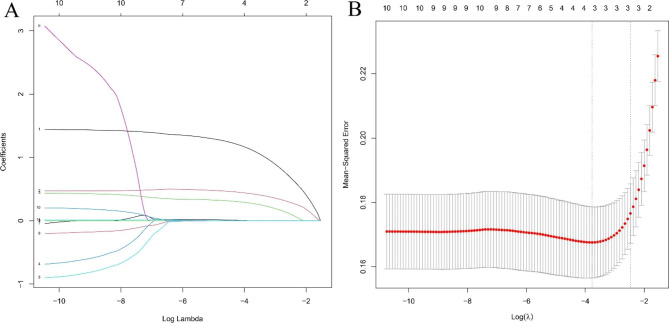
LASSO regression model screening risk factors *Variable selection by the LASSO regression model. A coefficient profile plot was constructed against the log(lambda) sequence. A: Eleven variables with nonzero coefficients were selected by deriving the optimal lambda. Choice of the optimal parameter (λ) in the LASSO regression model with logλ as the horizontal coordinate and regression coefficients as the vertical coordinate; B: Plot of λ versus number of variables with logλ as the bottom horizontal coordinate, model error value as the vertical coordinate, and number of variables as the top horizontal coordinate.*

The variables were screened by LASSO regression, the λ was taken when the model error was minimized, and three indicators were finally screened, namely TG/HDL-c, TC/HDL-c, and LDL-c/HDL-c, as shown in Fig. 2; Table 2. Collinearity diagnostics revealed no significant covariance among the screened indicators.

**Table 2:** Risk factors of the prediction model for obese children and adolescents

	β	SE	OR(95%CI)	*P*	VIF
Constant	-4.571	0.505	0.010	<0.001	
TG/HDL-c	1.378	0.293	3.967(2.234–7.044)	<0.001	1.412
TC/HDL-c	0.501	0.166	1.650(1.191–2.287)	0.003	2.271
LDL-c/HDL-c	0.357	0.242	1.429(0.889–2.298)	0.140	2.243

*OR: Odds Ratio; SE: Standard error; VIF: Variance Inflation Factor.*

### Diagnostic performance of TG/HDL-c, TC/HDL-c, LDL-c/HDL-c, and multi-indicator model

**Fig. 3:**
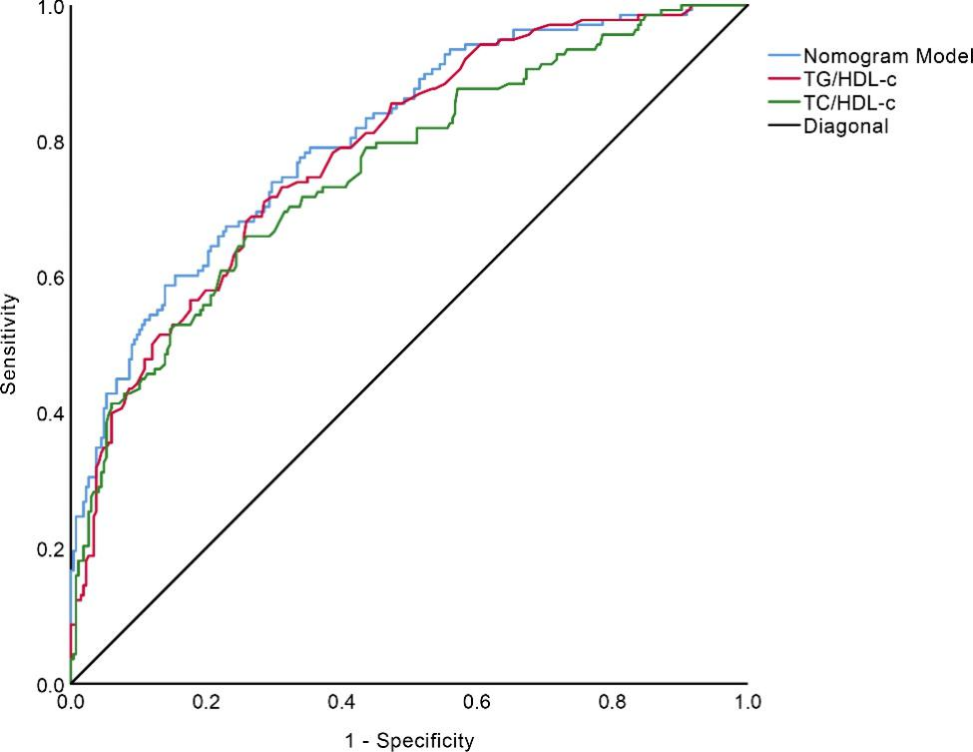
ROC curves of the predictive model for risk of IR

According to the LASSO regression results, the ROC curves were plotted with single indicators TG/HDL-c, TC/HDL-c, LDL-c/HDL-c, and multiple indicators (combined TG/HDL-c, TC/HDL-c, and LDL-c/HDL-c), as shown in Fig. 3. The comparison of the diagnostic value of the single and multiple indicator models is shown in Table 3. Multiple indicator models have better differentiation and specificity than single indicators model.

**Table 3:** Comparison of the diagnostic value of single-indicator and multi-indicator model

Model	AUC (95%CI)	SE	Sen	Sp	YI	AIC	BIC	PLR	NLR	PPV	NPV
TG/HDL-c	0.784 (0.738–0.830)	0.023	0.710	0.714	0.424	428.973	432.974	2.483	0.406	0.670	0.775
TC/HDL-c	0.757 (0.707–0.807)	0.026	0.659	0.741	0.400	434.464	438.465	2.544	0.460	0.619	0.772
LDL-c/HDL-c	0.734 (0.682–0.786)	0.026	0.457	0.902	0.359	449.310	455.311	4.663	0.602	0.516	0.765
Nomogram model	0.804 (0.760–0.849)	0.023	0.587	0.861	0.448	403.464	419.570	4.223	0.480	0.681	0.790

*AIC: Akaike Information Criterion; AUC: Area Under the Curve; BIC: Bayesian Information Criterion; CI: Confidence Interval; NLR: Negative Likelihood Ratio; Nomogram model: TG/HDL-c + TC/HDL-c + LDL-c/HDL-c; NPV: Negative Predicted Value; SE: Standard Error; Sen: Sensitivity; Sp: Specificity; PLR: Positive Likelihood Ratio; PPV: Positive Predicted Value; YI: Youden Index.*

### Development of IR-predicting nomogram

**Fig. 4:**
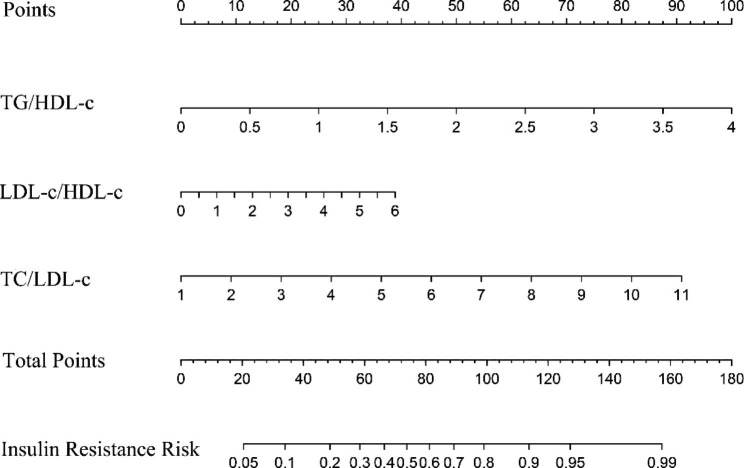
Nomogram model of the risk of developing IR

On the basis of the LASSO-logistic regression results, a nomogram model of the risk of IR in obese children and adolescents was developed, as shown in Fig. 4. The results of the nomogram model show that the scores increased with the TG/HDL-c, TC/HDL-c, and LDL-c/HDL-c values and led to an increase of the risk of developing IR.

To enhance the efficiency of clinical use, online dynamic software based on this line chart has also been developed (https://caoyx.shinyapps.io/dynnom/).

### Validation of nomogram model for IR

**Fig. 5:**
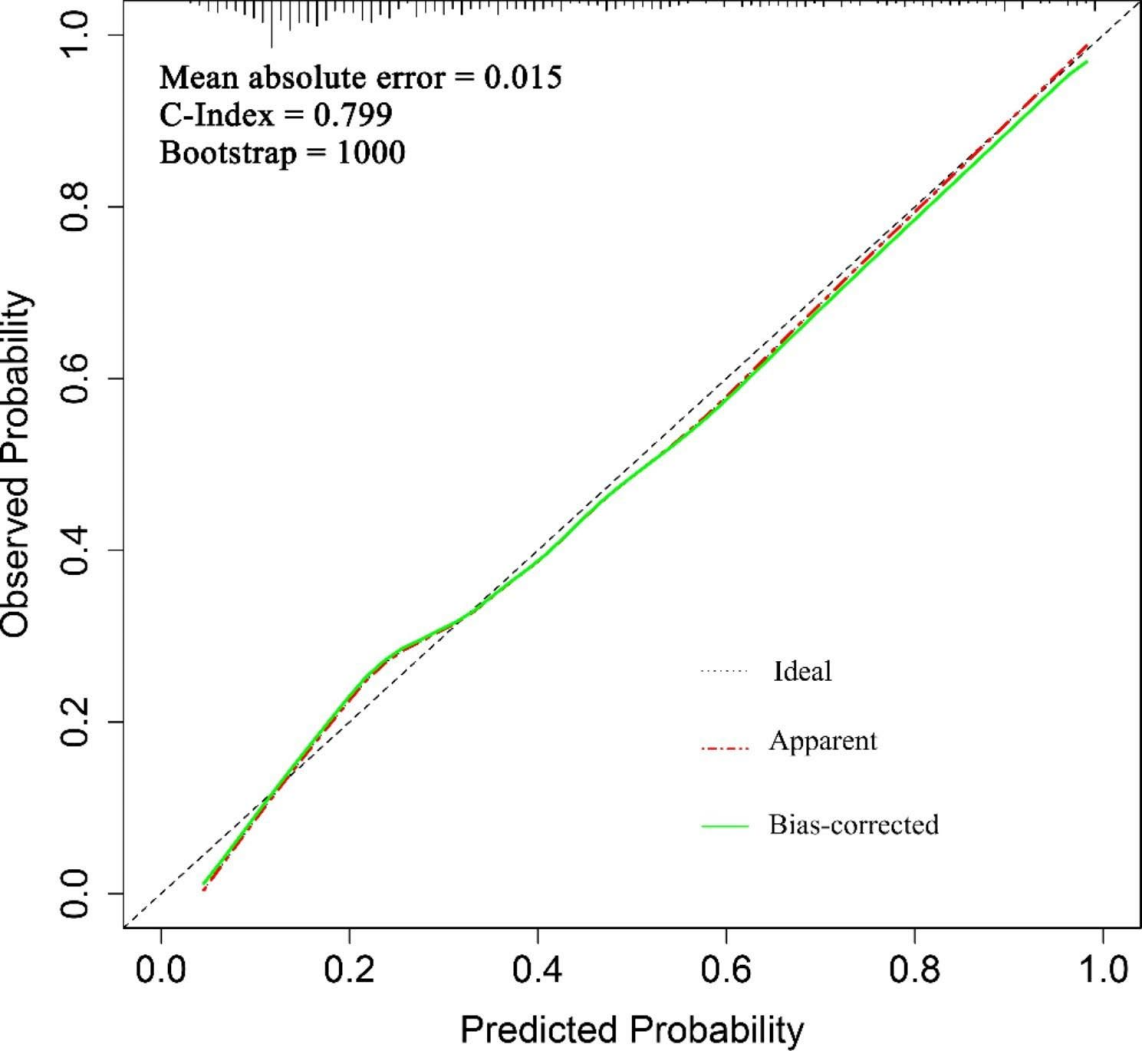
Calibration curve for internal validation of the nomogram model *Calibration plot of the nomogram. The diagonal dotted line showed an ideal model for the perfect prediction ability, the red line (Apparent line) represents the reality performance of the nomogram, and the green line (bias-corrected line). The closer fit to the diagonal dotted line, the better prediction ability of the nomogram.*

On the basis of the LASSO regression analysis results, the final indicators included in the IR risk prediction model were TG/HDL-c, TC/HDL-c, and LDL-c/HDL-c. The AUC of the model was 0.804 (95% CI: 0.760 to 0.849), as shown in Fig. 3. Repeating the Bootstrap self-sampling and internal validation 1000 times, the sampling results show a C-Index of 0.799, and the mean absolute error of the nomogram model was 0.015. After the Hosmer–Lemeshow goodness-of-fit test, the results indicate good model prediction (χ^2^ = 9.523, P = 0.300), as shown in Fig. 5.

### Decision curves for the IR-predicting nomogram model

**Fig. 6:**
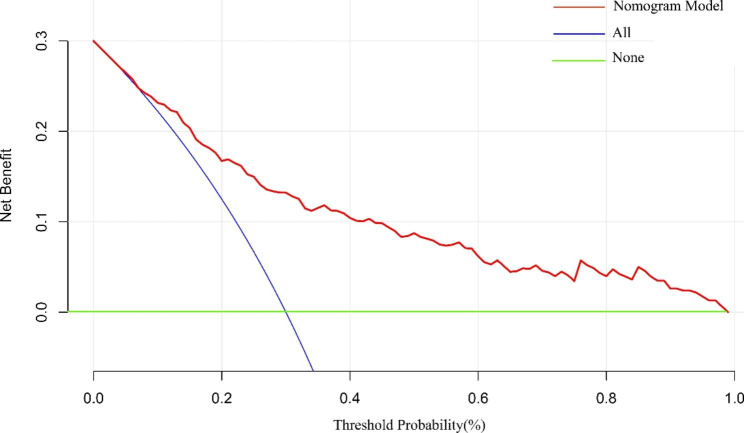
The decision curve analysis of the nomogram *Decision curve analysis for the nomogram. Notes: the y-axis measures the net benefit. The red line represents the IR risk nomogram. The blue line represents the assumption that all patients are IR. The green line represents the assumption that no patients was considered to exhibit IR. The decision curve showed that if the threshold probability of a patient was above 12%, using this IR nomogram in the current study to predict insulin resistance risk adds more benefit than all-interventionor the none-intervention.*

As shown in Fig. 6, when the risk of IR in obese children and adolescents was predicted using the nomogram model, the net benefit of applying the nomogram model was significantly higher than that of “all-intervention” or “no-intervention” when the risk was higher than 12%, indicating that the line graph has good clinical applicability.

## Discussion

Nomograms can transform complex regression equations into convenient and visual graphs, which have a high practical application[36]. Given that the anthropomorphology derived indicators included in this study were calculated based on height, weight, waist circumference, hip circumference, and blood lipids, the multiple covariance test revealed the covariance among indicators (VIF > 10), and LASSO regression was used to screen the indicators in this study to eliminate the adverse effects of the covariance among indicators on the prediction model and reduce the predictive efficacy. By using the LASSO regression method to construct a penalty function, insignificant functions can be compressed to zero, which is more effective than logistic regression, principal component analysis, and ridge regression in dealing with covariance parameters[37, 38]. In addition, the nomogram model after calibrated revealed good predictive accuracy between the acual and predicted probability (χ^2^ = 9.523, P = 0.300).

In this study, three lipid-derived indicators, TG/HDL-c, TC/HDL-c, and LDL-c/HDL-c, were finally selected and combined to build an IR-predicting nomogram model for obese children and adolescents, as we know this was the first published report. As an early symptomatic manifestation of IR [39] and a routine physical examination, blood lipids were easily obtained and have a strong practical and application value. For insulin, due to the unstability characteristic, blood sample collected for insulin measurement must be kept cold, immediately processed and plasma frozen as soon as possible [40], and another important reason was that measuring fasting insulin is not a routine test for Chinese obese children and adolescents.

Obesity leads to lipometabolic disturbance, such as elevated levels of TC, TG, and LDL-c and reduced levels of HDL-c[41], and also directly associated with IR[42]. When TG levels sustained chronically high may activated lipoprotein lipase and induced an increase in free fatty acid (FFA), ultimately leading to insulin sensitivity decreased[43]. The elevation of TG, TC, and LDL-c were risk factors for IR, while HDL-c was a protective factor[44]. Therefore, all these blood indicators are closely related to IR. Previous studies have shown that when used original blood profiles for IR prediction, the performance of the model was low [15], this means that the primary blood indicator are not reliable markers of insulin resistance. Li et al. [45] also found that TG/HDL-c was the best predictor of IR than that of TG or HDL-C alone. A combined lipid ratio may better reflect the overall interaction between lipid/lipoprotein fractions, and therefore associations with IR [46], and present less superposition of populations in predicted diseases [47]. In addition, elevated TG/HDL-c means more FFA transported to the liver for TG synthesis, which worsening of IR[48]. Therefore, the ratio of lipid profiles may be can used as predictors of IR[48].

HDL-c containing several subclasses, and small-sized HDL subclasses may contribute to increased risk of cardiovascular disease, whereas large-sized HDL subclasses are associated with decreased risk[49–51]. Studies found that when TG/HDL-c increased, the trend toward smaller HDL size was obvious, which indicated that the maturation of HDL might be impeded and the reverse cholesterol transport might be weakened[52], and this imbalance of the ratio may reveal the complexity of the metabolic processing.

Studies found that single lipid-derived indexes such as TC/HDL-c or TG/HDL-c could discriminate IR in children and adolescents [53, 54], but the efficiency was low. Ye et al.[55] found that an AUC of 0.66 (95% CI: 0.62 to 0.71) when a single TG/HDL-c predictive model was used and 0.71 (95% CI: 0.67 to 0.75) when TG/HDL-c was combined with the WC and the alanine transaminase. This result indicates that multi-indicator modeling has a higher diagnostic value than single-indicator modeling. In this study, the predictive efficacy was improved when the three derived metrics, TG/HDL-c, TC/HDL-c and LDL-c/HDL-c, were used for joint modeling. According to the results of AIC and BIC, we found that the goodness of fit of nomagram model was also better than signal lipid-derived indexes model.

In this study, only lipid-derived indicators were ultimately included, while none of the anthropometric-derived indicators were included. Several studies have found that anthropometric-derived indicators such as CI, WHtR, and BRI can be used as predictors of IR, and the participants of these studies included normal weight and obese children and adolescents[12–14]. The participants of this study were all obese children and adolescents, one-way ANOVA revealed that only four indicators, namely, AVI (P = 0.029), BMI (P = 0.038), WHtR (P = 0.040), and CI (P = 0.019), were significant difference between the IR and no-IR groups. And the AUC revealed that the diagnostic values were low (AUC < 0.600). Excess fat accumulation leads to an increase in FFA, mitochondrial fatty acid transport, and damage β-oxidation capacity, resulting in the inhibition of fat oxidation in the body[56], which finally leads to glucose tolerance impaired and the occurrence of IR[57]. This suggested that for obese children and adolescents, blood-related indicators maybe can more effectively reflect the evolution of IR. Therefore, in establishing the risk prediction model for the development of IR in obese children and adolescents, blood indicators should be the main focus[58, 59].

Usually, the steps for using the nomogram were as follows: (1) determine the participant’s value of each risk factor; (2) draw a straight line upward from each risk factor to the top point line; (3) find the sum of the points of all risk factors; (4) locate the sum on the total point reference line; (5) draw a straight line from total point line down to the bottom probability line to find the risk of IR. The operation was a bit cumbersome. To further enhance the clinical applicability of the nomogram model, a dynamic webpage was further developed to make the nomogram model more convenient in application.

## Limitations of the study

The three lipid-related indicators, namely, TG/HDL-c, TC/HDL-c, and LDL-c/HDL-c, were screened by LASSO-logistic regression, and the IR nomogram model for obese children and adolescents was constructed with a good predictive effect. However, this study has some limitations. (1) The prediction model was validate using the Bootstrap method, so the data collection from multicenter studies must be continued for external validation to further improve the model. (2) The anthropometric indices were calculated based on height, weight, waist circumference, and so on, which were not effectively incorporated in the final nomogram model. Therefore, in future studies, indicators related to body fat distribution can be introduced to build an improved model.

## Conclusion

In summary, IR is one of the fastest growing health challenges in the twenty-first century for obese children and adolescents, and exercise or other interventions can delay or prevent the occurrence and development of IR. Therefore, identifying high-risk groups is essential for the early initiation of health education and interventions.

The three early warning factors, namely, TG/HDL-c, TC/HDL-c, and LDL-c/HDL-c were screened by the LASSO-logistic regression combination, which could effectively establish a nomogram model for predicting the risk of IR in obese children and adolescents, and the diagnostic value of the combined model was better than that of the single indictor model. In conclusion, we recommend that Chinese obese children and adolescents can evaluate IR risk after routine physical examination, so that further health interventions can be taken as early as possible.

## Electronic supplementary material

Below is the link to the electronic supplementary material.

Supplementary Material 1

## Data Availability

The datasets for this study can get from corresponding author.

## Abbreviations

AVI: Abdominal volume index
BAI: Body Adiposity Index
BMI: body mass index
BRI: Body Roundness Index
BW: Body Weight
CI: Conicity Index
FPG: Fasting Plasma Glucose
FINs: Fasting Insulin
HOMA-IR: Homeostasis Model Assessment index of Insulin Resistance
HC: Hip Circumference
HDL-c: High-density Lipoprotein Cholesterol
IR: insulin resistance
LASSO: the least absolute shrinkage and selection operator
LDL-c/HDL-c: The ratio of LDL-c and HDL-c
WC: Waist Circumference
WHR: Waist Hip Rate
WHtR: Waist Height Rate
TC: Total cholesterol
TMI: Tri-Ponderal Mass Index
TG: Triglycerides
TG/HDL-c: The ratio of TG and HDL-c
TC/HDL-c: The ratio of TC and HDL-c.

## References

[1] Reaven GM (1988). Banting lecture 1988. Role of insulin resistance in human disease. Diabetes.

[2] Lee JM (2006). Insulin resistance in children and adolescents. Rev Endocr Metab Disord.

[3] Yin J, Li M, Xu L, Wang Y, Cheng H, Zhao X, Mi J (2013). Insulin resistance determined by Homeostasis Model Assessment (HOMA) and associations with metabolic syndrome among chinese children and teenagers. Diabetol Metab Syndr.

[4] Ormazabal V, Nair S, Elfeky O, Aguayo C, Salomon C, Zuñiga FA (2018). Association between insulin resistance and the development of cardiovascular disease. Cardiovasc Diabetol.

[5] Laakso M, Kuusisto J (2014). Insulin resistance and hyperglycaemia in cardiovascular disease development. Nat Rev Endocrinol.

[6] Goodwin PJ, Ennis M, Bahl M, Fantus IG, Pritchard KI, Trudeau ME, Koo J, Hood N (2009). High insulin levels in newly diagnosed breast cancer patients reflect underlying insulin resistance and are associated with components of the insulin resistance syndrome. Breast Cancer Res Treat.

[7] Argirion I, Weinstein SJ, Männistö S, Albanes D, Mondul AM (2017). Serum insulin, glucose, indices of insulin resistance, and risk of Lung Cancer. Cancer Epidemiol Biomarkers Prev.

[8] Copeland KC, Zeitler P, Geffner M, Guandalini C, Higgins J, Hirst K, Kaufman FR, Linder B, Marcovina S, McGuigan P (2011). Characteristics of adolescents and youth with recent-onset type 2 diabetes: the TODAY cohort at baseline. J Clin Endocrinol Metab.

[9] Yajnik CS, Katre PA, Joshi SM, Kumaran K, Bhat DS, Lubree HG, Memane N, Kinare AS, Pandit AN, Bhave SA (2015). Higher glucose, insulin and insulin resistance (HOMA-IR) in childhood predict adverse cardiovascular risk in early adulthood: the Pune Children’s study. Diabetologia.

[10] DeFronzo RA, Tobin JD, Andres R (1979). Glucose clamp technique: a method for quantifying insulin secretion and resistance. Am J Physiol.

[11] Matthews DR, Hosker JP, Rudenski AS, Naylor BA, Treacher DF, Turner RC (1985). Homeostasis model assessment: insulin resistance and beta-cell function from fasting plasma glucose and insulin concentrations in man. Diabetologia.

[12] Carneiro IB, Sampaio HA, Carioca AA, Pinto FJ, Damasceno NR (2014). [Old and new anthropometric indices as insulin resistance predictors in adolescents]. Arq Bras Endocrinol Metabol.

[13] Manios Y, Kourlaba G, Kafatos A, Cook TL, Spyridaki A, Fragiadakis GA (2008). Associations of several anthropometric indices with insulin resistance in children: the Children Study. Acta Paediatr.

[14] Li G, Wu HK, Wu XW, Cao Z, Tu YC, Ma Y, Li BN, Peng QY, Cheng J, Wu B (2019). The feasibility of two anthropometric indices to identify metabolic syndrome, insulin resistance and inflammatory factors in obese and overweight adults. Nutrition.

[15] He J, He S, Liu K, Wang Y, Shi D, Chen X (2014). The TG/HDL-C ratio might be a surrogate for insulin resistance in chinese nonobese women. Int J Endocrinol.

[16] Zhou M, Zhu L, Cui X, Feng L, Zhao X, He S, Ping F, Li W, Li Y (2016). The triglyceride to high-density lipoprotein cholesterol (TG/HDL-C) ratio as a predictor of insulin resistance but not of β cell function in a chinese population with different glucose tolerance status. Lipids Health Dis.

[17] Zhang L, Chen S, Deng A, Liu X, Liang Y, Shao X, Sun M, Zou H (2015). Association between lipid ratios and insulin resistance in a chinese population. PLoS ONE.

[18] Mostafa SA, Davies MJ, Morris DH, Yates T, Srinivasan BT, Webb D, Brady E, Khunti K (2012). The association of the triglyceride-to-HDL cholesterol ratio with insulin resistance in white european and south asian men and women. PLoS ONE.

[19] Pujos-Guillot E, Brandolini M, Pétéra M, Grissa D, Joly C, Lyan B, Herquelot É, Czernichow S, Zins M, Goldberg M (2017). Systems Metabolomics for Prediction of metabolic syndrome. J Proteome Res.

[20] Force GoCOT (2004). [Body mass index reference norm for screening overweight and obesity in chinese children and adolescents]. Zhonghua Liu Xing Bing Xue Za Zhi.

[21] Ricci MA, De Vuono S, Scavizzi M, Gentili A, Lupattelli G (2016). Facing morbid obesity: how to Approach it. Angiology.

[22] Kumar S, Kelly AS (2017). Review of childhood obesity: from epidemiology, etiology, and Comorbidities to Clinical Assessment and Treatment. Mayo Clin Proc.

[23] Ebbeling CB, Pawlak DB, Ludwig DS (2002). Childhood obesity: public-health crisis, common sense cure. Lancet.

[24] Harrell FE. Regression modeling strategies: with applications to linear models, logistic regression, and survival analysis. Volume 608. Springer; 2001.

[25] Haas JD, Campirano F (2006). Interpopulation variation in height among children 7 to 18 years of age. Food Nutr Bull.

[26] Censi L, Spinelli A, Roccaldo R, Bevilacqua N, Lamberti A, Angelini V, Nardone P, Baglio G (2014). Dressed or undressed? How to measure children’s body weight in overweight surveillance?. Public Health Nutr.

[27] Physical status (1995). The use and interpretation of anthropometry. Report of a WHO Expert Committee. World Health Organ Tech Rep Ser.

[28] Wang J, Thornton JC, Kolesnik S, Pierson RN (2000). Anthropometry in body composition. An overview. Ann N Y Acad Sci.

[29] Valdez R (1991). A simple model-based index of abdominal adiposity. J Clin Epidemiol.

[30] Thomas DM, Bredlau C, Bosy-Westphal A, Mueller M, Shen W, Gallagher D, Maeda Y, McDougall A, Peterson CM, Ravussin E (2013). Relationships between body roundness with body fat and visceral adipose tissue emerging from a new geometrical model. Obes (Silver Spring).

[31] Guerrero-Romero F, Rodríguez-Morán M (2003). Abdominal volume index. An anthropometry-based index for estimation of obesity is strongly related to impaired glucose tolerance and type 2 diabetes mellitus. Arch Med Res.

[32] Schulze MB, Thorand B, Fritsche A, Häring HU, Schick F, Zierer A, Rathmann W, Kröger J, Peters A, Boeing H (2012). Body adiposity index, body fat content and incidence of type 2 diabetes. Diabetologia.

[33] Peterson CM, Su H, Thomas DM, Heo M, Golnabi AH, Pietrobelli A, Heymsfield SB (2017). Tri-ponderal Mass Index vs Body Mass Index in estimating body Fat during Adolescence. JAMA Pediatr.

[34] Iasonos A, Schrag D, Raj GV, Panageas KS (2008). How to build and interpret a nomogram for cancer prognosis. J Clin Oncol.

[35] Kerr KF, Brown MD, Zhu K, Janes H (2016). Assessing the clinical impact of risk prediction models with decision curves: Guidance for correct interpretation and appropriate use. J Clin Oncol.

[36] Eastham JA, Kattan MW, Scardino PT (2002). Nomograms as predictive models. Semin Urol Oncol.

[37] Hu JY, Wang Y, Tong XM, Yang T (2021). When to consider logistic LASSO regression in multivariate analysis?. Eur J Surg Oncol.

[38] Friedman J, Hastie T, Tibshirani R (2010). Regularization Paths for generalized Linear Models via Coordinate Descent. J Stat Softw.

[39] Taskinen MR (2003). Diabetic dyslipidaemia: from basic research to clinical practice. Diabetologia.

[40] Farino ZJ, Morgenstern TJ, Vallaghe J, Gregor N, Donthamsetti P, Harris PE, Pierre N, Freyberg R, Charrier-Savournin F, Javitch JA (2016). Development of a Rapid insulin assay by homogenous time-resolved fluorescence. PLoS ONE.

[41] Klop B, Elte JWF, Cabezas MC (2013). Dyslipidemia in obesity: mechanisms and potential targets. Nutrients.

[42] Athyros VG, Doumas M, Imprialos KP, Stavropoulos K, Georgianou E, Katsimardou A, Karagiannis A (2018). Diabetes and lipid metabolism. Horm (Athens).

[43] Lewis GF, Carpentier A, Adeli K, Giacca A (2002). Disordered fat storage and mobilization in the pathogenesis of insulin resistance and type 2 diabetes. Endocr Rev.

[44] Baldeweg SE, Golay A, Natali A, Balkau B, Del Prato S, Coppack SW (2000). Insulin resistance, lipid and fatty acid concentrations in 867 healthy Europeans. European Group for the study of insulin resistance (EGIR). Eur J Clin Invest.

[45] Li C, Ford ES, Meng YX, Mokdad AH, Reaven GM (2008). Does the association of the triglyceride to high-density lipoprotein cholesterol ratio with fasting serum insulin differ by race/ethnicity?. Cardiovasc Diabetol.

[46] Millán J, Pintó X, Muñoz A, Zúñiga M, Rubiés-Prat J, Pallardo LF, Masana L, Mangas A, Hernández-Mijares A, González-Santos P (2009). Lipoprotein ratios: physiological significance and clinical usefulness in cardiovascular prevention. Vasc Health Risk Manag.

[47] Kinosian B, Glick H, Garland G (1994). Cholesterol and coronary heart disease: predicting risks by levels and ratios. Ann Intern Med.

[48] McLaughlin T, Reaven G, Abbasi F, Lamendola C, Saad M, Waters D, Simon J, Krauss RM (2005). Is there a simple way to identify insulin-resistant individuals at increased risk of cardiovascular disease?. Am J Cardiol.

[49] Barter PJ, Rye KA (1996). High density lipoproteins and coronary heart disease. Atherosclerosis.

[50] Superko RH (2001). Lipoprotein subclasses and atherosclerosis. Front Biosci.

[51] von Eckardstein A, Huang Y, Assmann G (1994). Physiological role and clinical relevance of high-density lipoprotein subclasses. Curr Opin Lipidol.

[52] Jia L, Long S, Fu M, Yan B, Tian Y, Xu Y, Gou L (2006). Relationship between total cholesterol/high-density lipoprotein cholesterol ratio, triglyceride/high-density lipoprotein cholesterol ratio, and high-density lipoprotein subclasses. Metabolism.

[53] García AG, Urbina Treviño MV, Villalpando Sánchez DC, Aguilar CA (2019). Diagnostic accuracy of triglyceride/glucose and triglyceride/HDL index as predictors for insulin resistance in children with and without obesity. Diabetes Metab Syndr.

[54] Giannini C, Santoro N, Caprio S, Kim G, Lartaud D, Shaw M, Pierpont B, Weiss R (2011). The triglyceride-to-HDL cholesterol ratio: association with insulin resistance in obese youths of different ethnic backgrounds. Diabetes Care.

[55] Chiang J-K, Lai N-S, Chang J-K, Koo M (2011). Predicting insulin resistance using the triglyceride-to-high-density lipoprotein cholesterol ratio in taiwanese adults. Cardiovasc Diabetol.

[56] Fritzen AM, Lundsgaard AM, Kiens B (2020). Tuning fatty acid oxidation in skeletal muscle with dietary fat and exercise. Nat Rev Endocrinol.

[57] Lundsgaard AM, Fritzen AM, Nicolaisen TS, Carl CS, Sjøberg KA, Raun SH, Klein AB, Sanchez-Quant E, Langer J, Ørskov C (2020). Glucometabolic consequences of acute and prolonged inhibition of fatty acid oxidation. J Lipid Res.

[58] Bastard JP, Lavoie ME, Messier V, Prud’homme D, Rabasa-Lhoret R (2012). Evaluation of two new surrogate indices including parameters not using insulin to assess insulin sensitivity/resistance in non-diabetic postmenopausal women: a MONET group study. Diabetes Metab.

[59] Disse E, Bastard JP, Bonnet F, Maitrepierre C, Peyrat J, Louche-Pelissier C, Laville M (2008). A lipid-parameter-based index for estimating insulin sensitivity and identifying insulin resistance in a healthy population. Diabetes Metab.

