# Supplementary material for: Nomogram model for the risk of insulin resistance in obese children and adolescents based on anthropomorphology and lipid derived indicators
Source: BMC Public Health. 2023 Feb 7;23:275. doi: 10.1186/s12889-023-15181-1 (PMC9906839; doi:10.1186/s12889-023-15181-1)
Supplement: Supplementary file 1 — Supplementary Material 1 [file 12889_2023_15181_MOESM1_ESM.docx]

Table S1 Calculation of anthropomorphology derived indicators

| No. | Derived Indicators | Indicators Sources |
| --- | --- | --- |
| 1 | BMI | BW(kg)/H(m)^2^ |
| 2 | WHR | WC(cm)/HC(cm) |
| 3 | WHtR | WC(cm)/H(cm) |
| 4 | CI(1) | WC(m)/[0.109×(BW(kg)/H(m))^0.5^] |
| 5 | BRI(2) | 364.2−365.5×(1–[(WC(m)/2π)/(0.5×H(m))]^2^)^0.5^ |
| 6 | AVI(3) | [2WC(cm)^2^+0.7(WC(cm)-H(cm))^2^]/1000 |
| 7 | BAI(4) | HC(cm)/H(m)^1.5^−18 |
| 8 | TMI(5) | BW(kg)/H(m)^3^ |

*AVI:* *Abdominal volume index; BAI:* *Body Adiposity Index; BMI:* *Body Mass Index; BRI:* *Body Roundness Index; BW: Body Weight; CI: Conicity index; H: Height; HC: hip circumference; WC: Waist Circumference; WHR:* *Waist Hip Rate; WHtR:* *Waist Height ratio; TMI:* *Tri-Ponderal Mass Index.*

**Reference**

1. Valdez R. A simple model-based index of abdominal adiposity. J Clin Epidemiol. 1991;44(9):955-6.

2. Thomas DM, Bredlau C, Bosy-Westphal A, Mueller M, Shen W, Gallagher D, et al. Relationships between body roundness with body fat and visceral adipose tissue emerging from a new geometrical model. Obesity (Silver Spring). 2013;21(11):2264-71.

3. Guerrero-Romero F, Rodríguez-Morán M. Abdominal volume index. An anthropometry-based index for estimation of obesity is strongly related to impaired glucose tolerance and type 2 diabetes mellitus. Arch Med Res. 2003;34(5):428-32.

4. Schulze MB, Thorand B, Fritsche A, Häring HU, Schick F, Zierer A, et al. Body adiposity index, body fat content and incidence of type 2 diabetes. Diabetologia. 2012;55(6):1660-7.

5. Peterson CM, Su H, Thomas DM, Heo M, Golnabi AH, Pietrobelli A, et al. Tri-Ponderal Mass Index vs Body Mass Index in Estimating Body Fat During Adolescence. JAMA Pediatr. 2017;171(7):629-36.
